# Supplementary material for: Modeling glioblastoma heterogeneity as a dynamic network of cell states
Source: Mol Syst Biol. 2021 Sep 16;17(9):e10105. doi: 10.15252/msb.202010105 (PMC8444284; doi:10.15252/msb.202010105)
Supplement: Supplementary file 6 — Source Data for Figure 5 [file MSB-17-e10105-s004.zip › Figure5A_sourcedata/GSEA_3071/hallmarks_stateIV.GseaPreranked.1623742551702/HALLMARK_TNFA_SIGNALING_VIA_NFKB.html]

Details for gene set HALLMARK\_TNFA\_SIGNALING\_VIA\_NFKB[GSEA]

|  || Dataset | state53071 |
| Phenotype | NoPhenotypeAvailable |
| Upregulated in class | na\_neg |
| GeneSet | HALLMARK\_TNFA\_SIGNALING\_VIA\_NFKB |
| Enrichment Score (ES) | -0.35479295 |
| Normalized Enrichment Score (NES) | -1.8809197 |
| Nominal p-value | 0.0 |
| FDR q-value | 0.004697924 |
| FWER p-Value | 0.061 |
Table: GSEA Results Summary

  

Fig 1: Enrichment plot: HALLMARK\_TNFA\_SIGNALING\_VIA\_NFKB      
 Profile of the Running ES Score & Positions of GeneSet Members on the Rank Ordered List

  

| PROBE | GENE SYMBOL | GENE\_TITLE | RANK IN GENE LIST | RANK METRIC SCORE | RUNNING ES | CORE ENRICHMENT || 1 | TNC |  |  | 66 | 1.751 | 0.0092 | No |
| 2 | FJX1 |  |  | 92 | 1.565 | 0.0239 | No |
| 3 | NINJ1 |  |  | 95 | 1.553 | 0.0430 | No |
| 4 | ID2 |  |  | 149 | 1.378 | 0.0500 | No |
| 5 | PLPP3 |  |  | 198 | 1.264 | 0.0566 | No |
| 6 | BTG2 |  |  | 200 | 1.260 | 0.0721 | No |
| 7 | LITAF |  |  | 252 | 1.144 | 0.0766 | No |
| 8 | G0S2 |  |  | 370 | 0.942 | 0.0658 | No |
| 9 | PMEPA1 |  |  | 373 | 0.939 | 0.0772 | No |
| 10 | TGIF1 |  |  | 402 | 0.906 | 0.0831 | No |
| 11 | LAMB3 |  |  | 413 | 0.892 | 0.0924 | No |
| 12 | PNRC1 |  |  | 481 | 0.830 | 0.0898 | No |
| 13 | PDE4B |  |  | 519 | 0.798 | 0.0927 | No |
| 14 | FOS |  |  | 530 | 0.786 | 0.1006 | No |
| 15 | B4GALT5 |  |  | 541 | 0.774 | 0.1084 | No |
| 16 | DUSP4 |  |  | 553 | 0.761 | 0.1157 | No |
| 17 | TSC22D1 |  |  | 588 | 0.735 | 0.1184 | No |
| 18 | KDM6B |  |  | 824 | 0.591 | 0.0805 | No |
| 19 | SAT1 |  |  | 859 | 0.575 | 0.0811 | No |
| 20 | SNN |  |  | 877 | 0.569 | 0.0850 | No |
| 21 | BCL6 |  |  | 921 | 0.552 | 0.0836 | No |
| 22 | CCNL1 |  |  | 1006 | 0.519 | 0.0739 | No |
| 23 | VEGFA |  |  | 1010 | 0.518 | 0.0798 | No |
| 24 | ZBTB10 |  |  | 1027 | 0.513 | 0.0831 | No |
| 25 | BTG1 |  |  | 1123 | 0.486 | 0.0709 | No |
| 26 | CSF1 |  |  | 1189 | 0.469 | 0.0642 | No |
| 27 | IRS2 |  |  | 1218 | 0.458 | 0.0646 | No |
| 28 | CFLAR |  |  | 1341 | 0.430 | 0.0464 | No |
| 29 | TUBB2A |  |  | 1373 | 0.422 | 0.0457 | No |
| 30 | NFE2L2 |  |  | 1626 | 0.363 | 0.0017 | No |
| 31 | DENND5A |  |  | 1673 | 0.355 | -0.0027 | No |
| 32 | MARCKS |  |  | 1713 | 0.348 | -0.0059 | No |
| 33 | PTGER4 |  |  | 1797 | 0.331 | -0.0178 | No |
| 34 | RCAN1 |  |  | 1802 | 0.331 | -0.0144 | No |
| 35 | BCL3 |  |  | 2125 | 0.280 | -0.0729 | No |
| 36 | PTPRE |  |  | 2187 | 0.269 | -0.0813 | No |
| 37 | CD44 |  |  | 2197 | 0.268 | -0.0797 | No |
| 38 | IL15RA |  |  | 2354 | -0.253 | -0.1066 | No |
| 39 | CLCF1 |  |  | 2356 | -0.254 | -0.1036 | No |
| 40 | SERPINB8 |  |  | 2571 | -0.278 | -0.1414 | No |
| 41 | TNFAIP2 |  |  | 2625 | -0.285 | -0.1481 | No |
| 42 | RHOB |  |  | 2638 | -0.288 | -0.1468 | No |
| 43 | NFKBIA |  |  | 2721 | -0.296 | -0.1589 | No |
| 44 | JUN |  |  | 2785 | -0.304 | -0.1672 | No |
| 45 | PANX1 |  |  | 2800 | -0.306 | -0.1661 | No |
| 46 | ZC3H12A |  |  | 2810 | -0.307 | -0.1640 | No |
| 47 | TRIP10 |  |  | 2836 | -0.311 | -0.1649 | No |
| 48 | RELB |  |  | 2850 | -0.313 | -0.1635 | No |
| 49 | EHD1 |  |  | 2914 | -0.320 | -0.1716 | No |
| 50 | ATF3 |  |  | 2977 | -0.330 | -0.1795 | No |
| 51 | KLF4 |  |  | 3019 | -0.335 | -0.1832 | No |
| 52 | ABCA1 |  |  | 3078 | -0.342 | -0.1901 | No |
| 53 | SOCS3 |  |  | 3148 | -0.353 | -0.1990 | No |
| 54 | TNFAIP3 |  |  | 3248 | -0.368 | -0.2135 | No |
| 55 | KLF10 |  |  | 3298 | -0.375 | -0.2182 | No |
| 56 | YRDC |  |  | 3376 | -0.387 | -0.2282 | No |
| 57 | BIRC3 |  |  | 3382 | -0.388 | -0.2243 | No |
| 58 | GEM |  |  | 3609 | -0.430 | -0.2625 | No |
| 59 | MAFF |  |  | 3620 | -0.432 | -0.2590 | No |
| 60 | MAP2K3 |  |  | 3674 | -0.439 | -0.2638 | No |
| 61 | PPP1R15A |  |  | 3793 | -0.465 | -0.2807 | No |
| 62 | TANK |  |  | 3826 | -0.471 | -0.2810 | No |
| 63 | HBEGF |  |  | 3905 | -0.488 | -0.2899 | No |
| 64 | NFKB1 |  |  | 3989 | -0.507 | -0.2996 | No |
| 65 | RIPK2 |  |  | 4107 | -0.542 | -0.3153 | No |
| 66 | JAG1 |  |  | 4168 | -0.559 | -0.3199 | No |
| 67 | NFIL3 |  |  | 4309 | -0.600 | -0.3394 | No |
| 68 | GADD45A |  |  | 4373 | -0.626 | -0.3437 | No |
| 69 | ATP2B1 |  |  | 4385 | -0.631 | -0.3379 | No |
| 70 | SDC4 |  |  | 4412 | -0.641 | -0.3349 | No |
| 71 | TNIP1 |  |  | 4441 | -0.650 | -0.3322 | No |
| 72 | NAMPT |  |  | 4551 | -0.705 | -0.3444 | No |
| 73 | DNAJB4 |  |  | 4606 | -0.737 | -0.3456 | Yes |
| 74 | PDLIM5 |  |  | 4612 | -0.739 | -0.3373 | Yes |
| 75 | SLC2A3 |  |  | 4629 | -0.754 | -0.3310 | Yes |
| 76 | DUSP5 |  |  | 4750 | -0.828 | -0.3437 | Yes |
| 77 | IER5 |  |  | 4759 | -0.835 | -0.3348 | Yes |
| 78 | FOSL1 |  |  | 4761 | -0.836 | -0.3246 | Yes |
| 79 | EIF1 |  |  | 4822 | -0.889 | -0.3250 | Yes |
| 80 | CEBPB |  |  | 4835 | -0.902 | -0.3160 | Yes |
| 81 | LIF |  |  | 4841 | -0.907 | -0.3057 | Yes |
| 82 | GFPT2 |  |  | 4843 | -0.910 | -0.2945 | Yes |
| 83 | SPSB1 |  |  | 4858 | -0.923 | -0.2856 | Yes |
| 84 | MYC |  |  | 4883 | -0.947 | -0.2784 | Yes |
| 85 | SQSTM1 |  |  | 4929 | -0.994 | -0.2747 | Yes |
| 86 | KLF6 |  |  | 4953 | -1.024 | -0.2663 | Yes |
| 87 | TNFAIP6 |  |  | 4987 | -1.072 | -0.2592 | Yes |
| 88 | SPHK1 |  |  | 5040 | -1.169 | -0.2546 | Yes |
| 89 | ACKR3 |  |  | 5047 | -1.180 | -0.2410 | Yes |
| 90 | KLF2 |  |  | 5058 | -1.209 | -0.2278 | Yes |
| 91 | EDN1 |  |  | 5081 | -1.246 | -0.2165 | Yes |
| 92 | DUSP1 |  |  | 5090 | -1.265 | -0.2022 | Yes |
| 93 | GADD45B |  |  | 5099 | -1.292 | -0.1876 | Yes |
| 94 | IRF1 |  |  | 5126 | -1.345 | -0.1758 | Yes |
| 95 | TNFAIP8 |  |  | 5130 | -1.363 | -0.1593 | Yes |
| 96 | PHLDA2 |  |  | 5155 | -1.458 | -0.1457 | Yes |
| 97 | SERPINE1 |  |  | 5162 | -1.501 | -0.1281 | Yes |
| 98 | ETS2 |  |  | 5200 | -1.662 | -0.1144 | Yes |
| 99 | PLAUR |  |  | 5213 | -1.721 | -0.0952 | Yes |
| 100 | IL18 |  |  | 5224 | -1.814 | -0.0745 | Yes |
| 101 | F3 |  |  | 5230 | -1.856 | -0.0522 | Yes |
| 102 | PTX3 |  |  | 5262 | -2.327 | -0.0291 | Yes |
| 103 | INHBA |  |  | 5280 | -2.740 | 0.0019 | Yes |
Table: GSEA details [plain text format]

  

Fig 2: HALLMARK\_TNFA\_SIGNALING\_VIA\_NFKB: Random ES distribution      
 Gene set null distribution of ES for **HALLMARK\_TNFA\_SIGNALING\_VIA\_NFKB**
